# Supplementary figures and images for: Effects of fermented feeds and ginseng polysaccharides on the intestinal morphology and microbiota composition of Xuefeng black-bone chicken
Source: PLoS One. 2020 Aug 11;15(8):e0237357. doi: 10.1371/journal.pone.0237357 (PMC7418966; doi:10.1371/journal.pone.0237357)

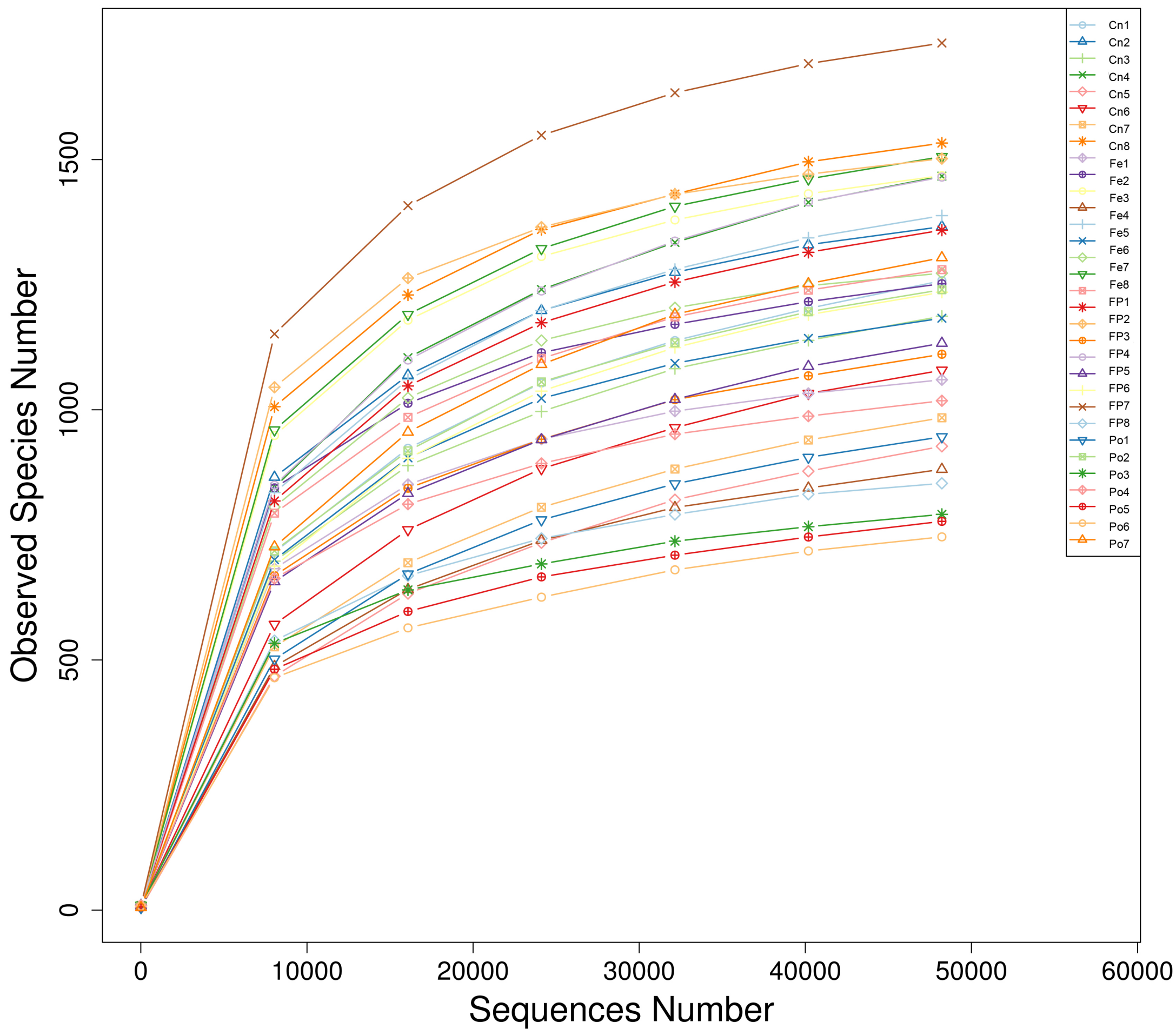

Observed species

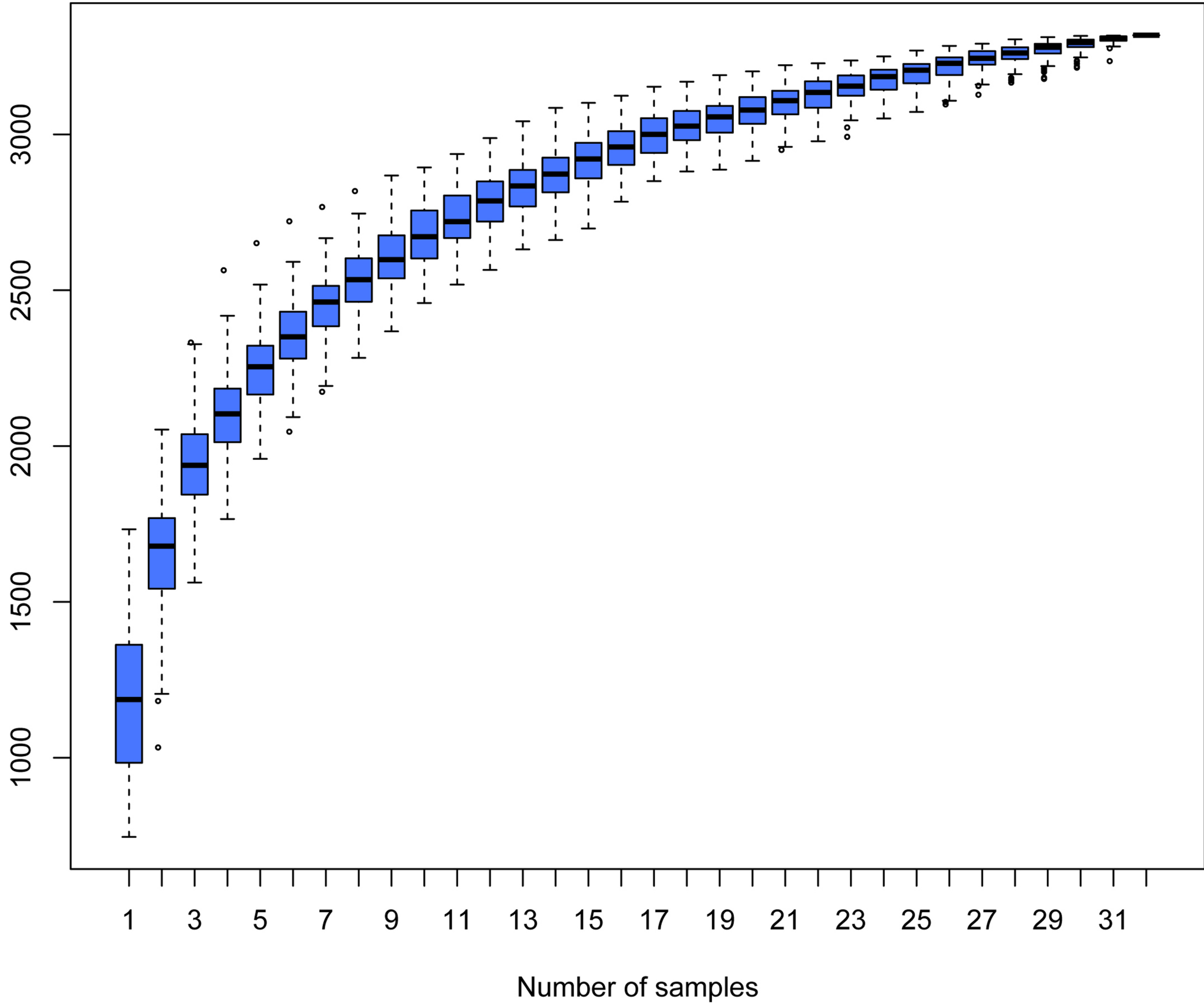

Supplement: S1 Fig — Number of sequences (A) and number of sample (B) rarefaction curves for the sampled jejunum microbiotas. Number of detected OTUs on the y-axis; number of sequences (A) and of samples (B) on the x-axis. (PDF) [file pone.0237357.s001.pdf]

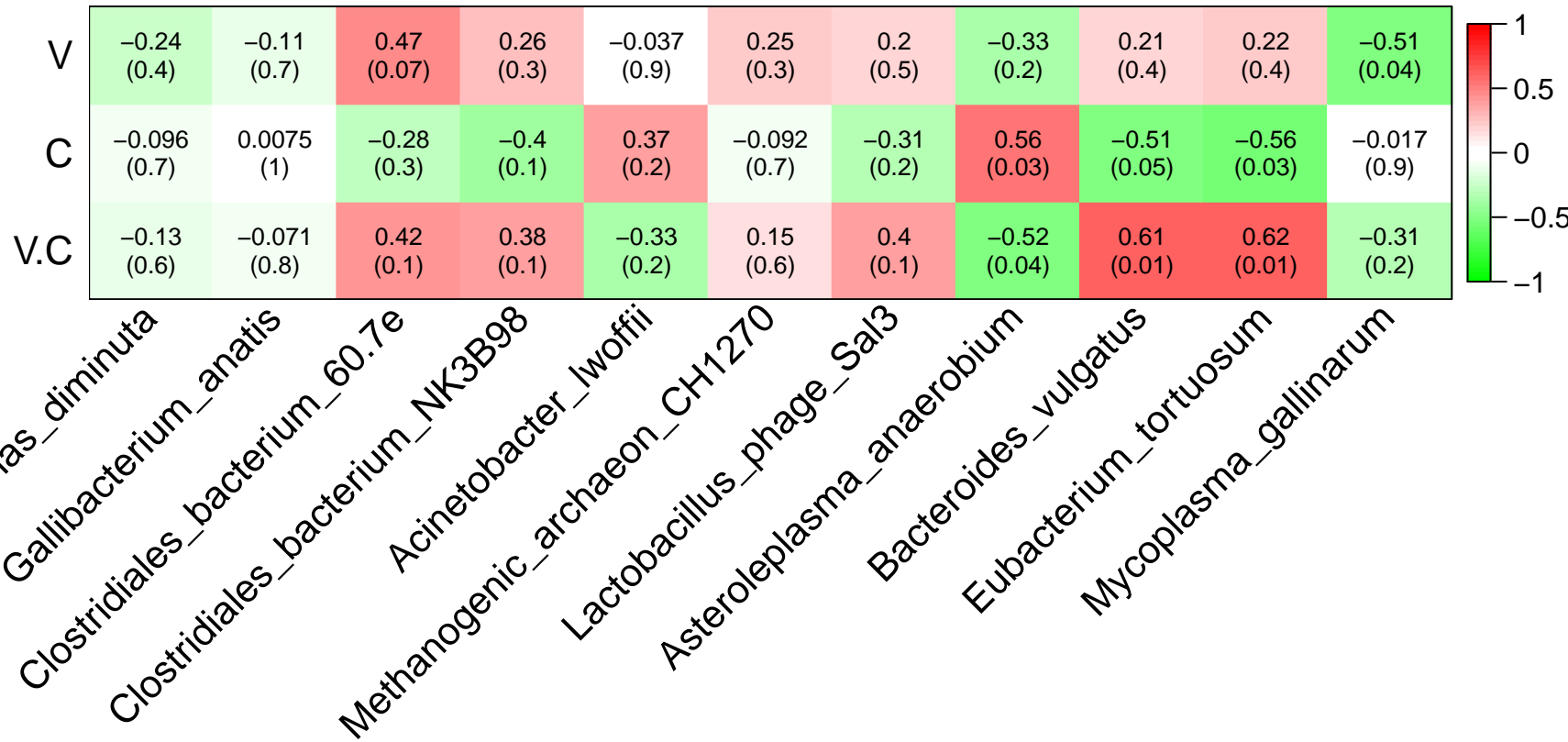

Supplement: S2 Fig — (PDF) [file pone.0237357.s002.pdf]
