# Supplementary material for: Effects of fermented feeds and ginseng polysaccharides on the intestinal morphology and microbiota composition of Xuefeng black-bone chicken
Source: PLoS One. 2020 Aug 11;15(8):e0237357. doi: 10.1371/journal.pone.0237357 (PMC7418966; doi:10.1371/journal.pone.0237357)
Supplement: S1 Table — (DOCX) [file pone.0237357.s003.docx]

**S1 Table Basic diet formulations and nutrient composition (%)**

| **Ingredient** | **Content (%)** | |
| --- | --- | --- |
|  | **0 to 4 weeks** | **5 to 22 weeks** |
| Corn | 59.0 | 59.0 |
| Soybean meal | 20.0 | 17.2 |
| Cottonseed meal | 3.8 | 8.5 |
| Peanut meal | 2.2 |  |
| Canola meal | 4.0 | 2.3 |
| Fish meal | 4.1 | 2.0 |
| Wheat bran | 0.6 | 5.0 |
| Rapeseed oil | 1.0 | 0 |
| Calciumhy droxide | 0.8 | 0.6 |
| Limestone powder | 0.5 | 0.8 |
| Premix^1^ | 4.5 | 4.5 |
| **Nutritional level** |  |  |
| Metabolizable energy（MJ/kg） | 12.46 | 12.34 |
| Crude protein | 20.55 | 19.42 |
| Available phosphorus | 0.45 | 0.4 |
| Methionine | 0.51 | 0.42 |
| Lysine | 1.1 | 1.11 |

^1^Premix provided per kg of diet: Fe ≥1000 mg; Cu ≥200 mg; Mn ≥1000 mg; Zn ≥1000 mg; I ≥15 mg; Se ≥2.5mg; Ca ≥50g；Total phosphorus ≥2.5%；4%≤ Salt ≤10%; Vitamin A ≥10000 IU; Vitamin D_3_ ≥3000 IU; Vitamin E ≥30 IU; Vitamin B_1_ ≥30 mg; Vitamin B_2_ ≥130 mg; Vitamin B_6_ ≥60 mg; Vitamin B_12_ ≥0.3 mg; Thiamine ≥30mg; Riboflavin ≥130 mg; [Niacin](javascript:;) ≥600 mg; Choline chloride ≥7500 mg; Calcium pantothenate ≥45 mg; Folic acid ≥22 mg; phytases ≥4000U.
